# Applying Self-Supervised Learning to Image Quality Assessment in Chest CT Imaging

**DOI:** 10.3390/bioengineering11040335

**Published:** 2024-03-29

**Authors:** Eléonore Pouget, Véronique Dedieu

**Affiliations:** 1 Department of Medical Physics, Jean Perrin Comprehensive Cancer Center, F-63000 Clermont-Ferrand, France; veronique.dedieu@clermont.unicancer.fr; 2 UMR 1240 INSERM IMoST, University of Clermont-Ferrand, F-63000 Clermont-Ferrand, France

**Keywords:** self-supervised learning, feature representation learning, convolutional denoising autoencoder, task-based approach, model observer, chest CT image

## Abstract

Many new reconstruction techniques have been deployed to allow low-dose CT examinations. Such reconstruction techniques exhibit nonlinear properties, which strengthen the need for a task-based measure of image quality. The Hotelling observer (HO) is the optimal linear observer and provides a lower bound of the Bayesian ideal observer detection performance. However, its computational complexity impedes its widespread practical usage. To address this issue, we proposed a self-supervised learning (SSL)-based model observer to provide accurate estimates of HO performance in very low-dose chest CT images. Our approach involved a two-stage model combining a convolutional denoising auto-encoder (CDAE) for feature extraction and dimensionality reduction and a support vector machine for classification. To evaluate this approach, we conducted signal detection tasks employing chest CT images with different noise structures generated by computer-based simulations. We compared this approach with two supervised learning-based methods: a single-layer neural network (SLNN) and a convolutional neural network (CNN). The results showed that the CDAE-based model was able to achieve similar detection performance to the HO. In addition, it outperformed both SLNN and CNN when a reduced number of training images was considered. The proposed approach holds promise for optimizing low-dose CT protocols across scanner platforms.

## 1. Introduction

Many new computed tomography (CT) reconstruction techniques have been developed in recent years to address the concern of reducing radiation exposure levels [1]. These methods typically exhibit nonlinear properties, which can result in an unfamiliar image texture in very low-dose CT [2]. As traditional physical metrics may not be adequate to assess image quality in nonlinear reconstructions, a task-based paradigm assessment has been advocated [3,4]. For signal detection tasks, the Bayesian ideal observer is optimal but its computation is often analytically intractable [5,6]. As an alternative, the Hotelling observer (HO) is commonly employed. The HO is the ideal linear observer in the sense that it maximizes the signal–noise ratio of the test statistic [6]. Its definition requires inverting the covariance matrix, for which its computational cost makes the HO difficult to implement in clinical practice [7,8].

Recent efforts have demonstrated the ability of supervised learning-based approaches to produce accurate estimates of HO performance [3,6,9,10,11]. For example, in a preliminary work, Zhou et al. [6] investigated the use of simple linear single-layer neural networks (SLNNs) to approximate the HO test statistic for binary signal detection tasks. They showed that this learning-based method was able to produce accurate estimates of the HO performance. More recently, Kim et al. [3] have demonstrated the feasibility of using convolutional neural networks (CNNs) to provide similar detection performance to the HO in breast CT images. The ability of CNNs to learn sophisticated image representations directly from the image data makes CNNs powerful tools in medical image analysis [12,13]. The accuracy and robustness of such a supervised approach, however, are dependent on the availability of large-scale annotated images [10,13,14]. Such labelled datasets are difficult to obtain in the medical domain due to the complexity of manual annotation and inter- and intra-observer variability [13,14].

In response to this challenge, various methods have been proposed. One approach relies on the concept of transfer learning, whereby general features are extracted from different domains in which large labeled datasets are available [12,13,14]. However, this approach is often unable to extract discriminative features for medical image analysis problems [12,13,14]. Another approach is based on self-supervised learning (SSL), which plays a growing role in the field of machine learning due to its capability of learning representations that are transferable to different downstream tasks [15,16]. This learning paradigm reduces the reliance on labeled data by training a model to extract meaningful representations of the input data with no manual labeling required [16]. Recent studies have leveraged the potential of SSL for various tasks such as image segmentation and classification [15,16,17]. The success of this approach strongly depends on how the pretext tasks are chosen [15]. Among the pretext tasks proposed in the literature, the category of pixel-to-pixel follows the paradigm of generating low-dimensional representations of inputs for use in downstream tasks [15].

In this context, autoencoders (AEs) and extensions such as Sparse AE [18], Denoising AE (DAE) [19,20], or Convolutional AE (CAE) have gained wide popularity because of their ability to extract discriminative and robust features [12,15,21,22,23,24]. The use of deep learning techniques in a preprocessing step allows shallow machine learning algorithms, such as support vector machine (SVM), to be used to interpret encoded features for classification [21,22]. This framework has been successfully applied in various domain applications [21,22,25,26]. In particular, CAEs have performed well in medical image classification [12,13].

This study proposes an SSL-based model observer to predict HO performances in very low-dose chest CT images. To the best of our knowledge, this is the first study to apply the SSL framework for designing a mathematical model observer. We hypothesize that integrating the SSL paradigm into model observer pipelines holds promise for implementing the task-based optimization of CT systems. Our approach combined CAE for the feature representation and SVM for the classification task. We compared this approach to state-of-the-art supervised methods through different signal detection tasks of varying difficulty. Computer-based simulations were conducted by addressing different noise structures to investigate the generalization property of the proposed SSL-based model observer. This property is a key factor in ensuring equivalent image quality across a fleet of scanners with increasing differences between reconstruction techniques.

## 2. Materials and Methods

### 2.1. Data

The dataset used in this work comprised images generated from the Lungman multipurpose chest phantom that was reconstructed with various regularization terms proposed for low-dose CT [27]. This dataset was constructed from computer-based simulations, as described in a previous work [28]. The latter showed that the generalization performance of an observer strongly depends on the ability of the feature extractor to filter the noise out and reveal the structures of the original data. We proposed here an extension of this work by investigating the benefit of using deep learning techniques as feature extractors in various noise structures.

To evaluate the generalization properties of the proposed SSL-based model observer, we conducted signal detection tasks over the whole dataset regardless of the regularization strategy in order to be representative of the practical use of the numerical observers [29].

**Figure 1:**
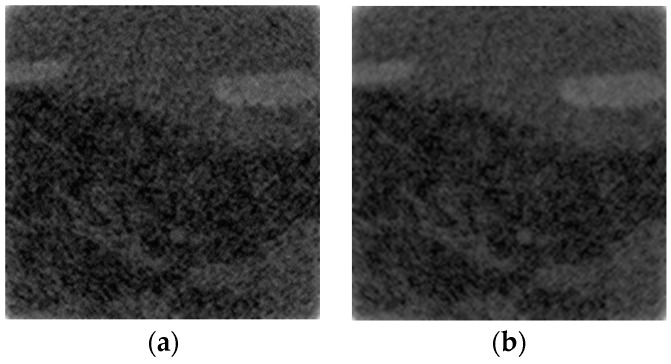
CT images of the Lungman phantom using (**a**) the Green and (**b**) the quadratic potential function into the regularization term of the iterative reconstruction method employed.

To get as close as possible to clinical tasks, all simulations addressed background-known-statistically (BKS) signal detection tasks [5]. The image data were generated by extracting 64 × 64 lung anthropomorphic textures from the simulated images, as showed in Figure 1. To generate the signal-present images, two signal objects were modeled as detailed below.

#### 2.1.1. Signal Known Exactly (SKE)

The first signal detection task employed a nodule signal profile given by the following [30,31]:

(1) $$f_{s}=\left\{\begin{array}{cc} A_{s}{\left[1-{\left(\frac{\left|r_{m}-r_{c}\right|}{R}\right)}^{2}\right]}^{z} & ,\;\left|r_{m}-r_{c}\right|\leq R\;\\ 0 & ,\;otherwise \end{array}\right.$$

where $\left|r_{m}-r_{c}\right|$ is the distance between the location of the *m*th pixel and the signal center, *R* is the signal radius, and *A_s_* is the parameter that controls the signal amplitude. The parameters were set as follows: *R* was fixed at 4 mm, *z* was fixed at 4, and signal amplitude was set to −870 HU, mimicking the attenuation of low-attenuation ground-glass opacities (GGO) [32]. In this task, the signal was invariant, referring to the signal-known-exactly (SKE) paradigm.

#### 2.1.2. Signal Known Statistically (SKS)

For the second signal detection task, we modeled an elliptical profile given by the following:

(2) $$f_{s}=A_{s}\exp\left({-(R_{\Phi }\left(r_{m}-r_{c}\right))}^{T}D^{-1}(R_{\Phi }\left(r_{m}-r_{c}\right))\right),$$

where D is a diagonal matrix that controls the width of the signal along each direction σ_x_ and σ_y_, and $R_{\theta }$ is the Euclidian rotation matrix that controls the angle of rotation for the signal, *Φ* [5,33]. In this study, we fixed σ_x_ = 5 and σ_y_ = 1.5, and *Φ* was randomly sampled from the set {0°, 45°, 90°, 135°} according to the detection task previously used by Granstedt et al. [5].

### 2.2. Hotelling Observer

The test statistic of a linear model observer, t, is computed by the following [3,5,6]:

(3) $$t(f)=w^{T}f,$$

where f is a column vector of the image, and w is the linear observer template [3]. The HO employs the population equivalent of the Fisher linear discriminant, which can be defined as follows [5,6]:

(4) $$w_{HO}={\left[\frac{1}{2}\left(K_{0}+K_{1}\right)\right]}^{-1}∆\bar{f},$$

where $K_{i}$ is the covariance matrix of the measured data under each hypothesis, and $∆\bar{f}$ is the mean difference between signal-present and signal-absent images [5,31]. The dimension of the covariance matrix is *M*^2^ where *M* is the number of image pixels [3]. An accurate estimate of *K* requires the collect of at least ten times *M*^2^ sample images which makes the HO estimation difficult if not impossible in practical usage [7].

In Equation (4), the inverse covariance matrix serves to decorrelate the noise in the image [31]. This point is particularly interesting to understand to what extent human observers are able to adapt to the statistical properties of the images managed by the reconstruction process, as previously stated by Abbey et al. [31].

### 2.3. Supervised Learning-Based Model Observer

Numerous works have successfully employed supervised learning-based technologies for implementing model observers [3,6,29,34,35]. In this work, a CNN and an SLNN were explored for binary signal detection tasks. The area under the receiver-operating-characteristic curve (AUC) was used for assessing the detection performance. The results obtained were compared to those produced by the proposed SSL-based model observer.

We optimized the structure of the CNN using brute-force searching in the parameter space defined by the depth (from 2 to 10) and the number of filters (4, 8, and 16). The filter size in each convolutional layer was set as 3 × 3 pixels in order to reduce the number of model parameters, as evoked by Kim et al. [3]. No downscaling layer was included to prevent the removal of any high-frequency component [3]. Each convolutional layer was followed by a LeakyReLU activation function to add nonlinear property into the network [3], while a sigmoid function was used after the fully connected layer. The output of the model can thus be interpreted as the posterior probability that an observation belongs to one of the two possible classes in a binary classification problem. In this work, the SLNN was not employed as a linear model and included the sigmoid function as the activation function.

The network training was performed in Tensorflow using a mini-batch stochastic gradient descent algorithm, i.e., the Adam algorithm [5,6,36,37], with 50 epochs to minimize the cross-entropy loss function. During the network training, the train–validation–test scheme [6] was used. We generated 5000 images to prepare the training dataset. Both the validation and testing datasets comprised 200 image pairs. The CNN resulting in the minimum validation cross-entropy was defined as the optimal model in the explored search space [6]. Once the network structure was optimized, the performance of the selected CNN was evaluated as a function of the training dataset size. This point is of particular interest as it represents the principal limitation of implementing CNNs in practical usage.

### 2.4. SSL-Based Model Observer

#### 2.4.1. AE

An AE is a specialized type of artificial neural network that consists of two components: the encoder and decoder. The first maps the input data vector *X* into a hidden representation *h*, while the latter aims to reconstruct *X* from the embedding *h*. This process can be formulated as follows (considering an AE with one hidden layer):

(5) $$\begin{array}{c} h=s_{1}(W_{1}X+b_{1}),\\ X^{\prime }=s_{2}(W_{2}h+b_{2}) \end{array}$$

where $s_{1}$ and $s_{2}\;$are activation functions. The AE is parametrized by the weight matrices *W*_1_ and *W*_2_ and bias vectors *b*_1_ and *b*_2_. Training an AE involves finding parameters θ = (*W*_1_, *W*_2_, *b*_1_, *b*_2_) since they minimize the reconstruction error on a given dataset [23,24]. The cross-entropy was used as the loss function as follows:

(6) $$L_{c}(W_{1},W_{2},b_{1},b_{2};X)=-\sum_{i=1}^{N}\left[X_{i}\log\left({X^{\prime }}_{i}\right)+\left(1-X_{i}\right)\log(1-{X^{\prime }}_{i})\right]$$

It is worthwhile noting that considering the mean-squared error as the loss function, the optimal linear one-layer AE projects the data onto a subspace defined by its principal directions [5]. Therefore, the principal component analysis (PCA) method can be thought of as a simplified form of AE. The PCA was performed in this work for comparison.

A weight-decay regularization term can be added to Equation (6) to limit the increase in weights that can occur during the training process, leading to model overfitting [22,23]. Thus, the objective function can be formulated as follows:

(7) $$L=L_{c}+\lambda W^{2},$$

where W^2^ guarantees the weight matrix having small elements. In this formulation, the penalty term is a quadratic constraint referring to l_2_-norm regularization. The hyper-parameter λ controls the strength of the regularization [23]. Classically, this parameter lies in the range [0, 0.1]. We varied the parameter λ over this interval to study its impact on the AE discriminative ability.

Another way to solve the overfitting issue is to consider DAE, which introduces noise in the original data. The DAE is trained to reconstruct the corrupted input, minimizing the loss function between the reconstructed *X′* and the original data *X* [23]. The corrupting operation was achieved by randomly setting some pixels in the input sample to zero, leading to more robust features than the traditional AE [19,20]. Compared to the other variants of the AE that ignore local connections between image content, convolutional AEs combine the advantage of a CNN and an AE to preserve spatial information, potentially limiting the redundancy in the parameters [13,22]. They have performed well in image denoising and classification [13], justifying their use in this work.

The structure of a convolutional DAE (CDAE) is similar to that of a DAE, except that it deals with convolutional layers rather than fully connected layers [38,39]. We optimized the structure of the network by performing a Bayesian search [38] in the parameter space defined by the depth (from 1 to 8), the number of filters (4, 8, or 16), and the number of latent units (from 2 to 32). Each convolutional layer comprised filters of 3 × 3 pixels and was followed by a LeakyReLU activation function to add nonlinear properties into the network. A sigmoid function was used after the last convolutional layer of the decoder. The output of the model was thus bounded on the interval [0, 1], which was of interest because the images were expressed in attenuation coefficients (cm^−1^). Zero padding was applied in order to maintain the size of the input. Compared to the traditional grid search method, which treats hyper-parameter sets independently, Bayesian optimization is an informed search method that learns from previous iterations [40,41,42]. As a result, this method can determine optimal hyper-parameters with fewer trials. This is of particular interest for optimizing network architecture where the complexity of grid search grows exponentially with the addition of new parameters. The Bayesian search was performed 20 times on 5000 images extracted from the whole dataset. For each trial, the training and testing sets were generated by a resampling procedure. During network training, the cross-entropy loss function was used, as well as the Adam optimizer [36]. An early-stopping strategy was employed, acting as the hyper-parameter for the number of epochs.

#### 2.4.2. SVM

For all learned features, the SVM was used for the classification task. This learning technique, based on a differentiable hinge loss, became popular due to its strong generalization capabilities. To achieve a non-linear separation between classes, a kernel function was introduced in the SVM formulation, including linear, polynomial, sigmoid, and RBF kernels [21]. The RBF function was employed in this study because of its ability to produce complex decision boundaries [40]. In this formulation, two hyper-parameters *C* and σ have to be adjusted. Parameter *C* controls the trade-off between correct classification on training samples and the maximization of the decision function’s margin, while σ defines the kernel width [43]. For example, the value of σ being too low makes the radius of influence of the support vector only include the support vector itself, which can lead to overfitting [43]. On the other hand, large values of σ can make the model too constrained [43].

The SVM performance strongly depends on the proper selection of these hyper-parameters. To do so, we used a grid search method by applying a five-fold cross-validation (CV) procedure. This strategy was justified by the fact that the search space was limited to only two parameters, *C* and σ. The AUC was used as the score metric for evaluating the quality of the model predictions. Compared to other metrics, the AUC does not require an optimizing threshold for each label. The selection of the optimal values of *C* and σ was carried out on 1000 images extracted from the image database that did not overlap with the images used for the CDAE network structure optimization. After the optimal model was selected, the performance of the SSL-based model observer was assessed as a function of the training dataset size.

## 3. Results

### 3.1. Optimal Structure of the CNN

**Figure 2:**
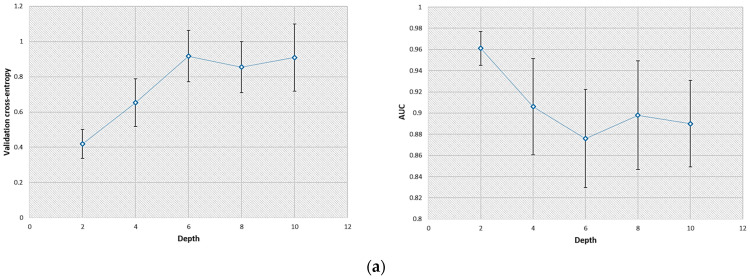

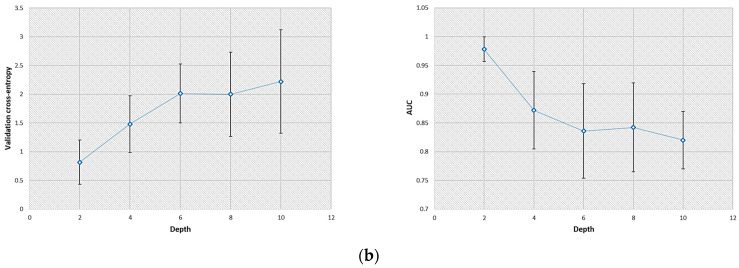
Effect of the convolutional layer number (from two to ten layers) on the CNN performance evaluated during network structure optimization. The error bars displayed correspond to the standard deviation obtained during this process. (**a**) Values of the validation cross-entropy (**left**) and AUC (**right**) for the SKE/BKS detection task; (**b**) values of the validation cross-entropy (**left**) and AUC (**right**) for the SKS/BKS detection task.

The train–validation–test scheme was repeated twenty times to improve the statistical reliability of the results. The validation cross-entropy, plotted in Figure 2, increased with the depth of the CNN for both tasks investigated. This result was most likely due to the overfitting issue that can occur when adding many hidden layers in the neural network.

When the SKE/BKS detection task was considered, the detection performance (i.e., AUC) evaluated on the testing dataset was 0.96 and 0.89 for the CNN with two and ten convolutional layers, respectively. These values were 0.98 and 0.82 for the SKS/BKS detection task. Therefore, the CNN that possessed two convolutional layers (each having eight filters) was selected for each detection task considered. It can be noted that the standard deviations associated with the results produced by the CNN increased with the convolutional layer number.

### 3.2. Optimal Parameters for the SSL-Based Model Observer

For both detection tasks, the optimal encoder derived from the Bayesian search comprised one convolutional layer with 16 filters, followed by a LeakyReLu activation function and finally a fully connected layer that processed the features maps into 25 latent units.

In SSL, the regularization hyper-parameter is one of the main parameters influencing the classification accuracy [21]. Table 1 shows how the hyper-parameter λ, which controls the relative importance of the weight-decay regularization applied during the CDAE training process, impacts the choice of *C* and σ in the SVM formulation.

It was observed that, as the value of λ increased, the classifier’s decision function favored low values of σ, translating interactions between similar observations. This can be explained by the fact that, for high values of λ, the weights learned converge towards zero. As a consequence, the encoder resembled a linear model, leading to underfitting situations. Conversely, low values of λ resulted in unregularized models, leading to the overfitting problem. Therefore, σ acts as a good structural regularizer for the SVM model.

From these results, we fixed the regularization parameter as λ = 0.001 for both the detection tasks considered. In this case, the optimal models are positioned on the diagonal of *C* and 1/σ. Taking intermediate values of σ and lowering the value of *C* seemed to be a good trade-off between generalization and prediction accuracy. However, lower *C* values generally relate to more support vectors, which can lead to a growing storage requirement and computational complexity, while high *C* values may increase the fitting time [21,43]. In this work, the model seemed to perform equally regardless of the value of *C* for intermediate values of σ. This reflected the fact that no more training points were located within the margin, and the parameter σ was acting alone as a good regularizer [43].

### 3.3. Detectability Comparison between Observers

**Figure 3:**
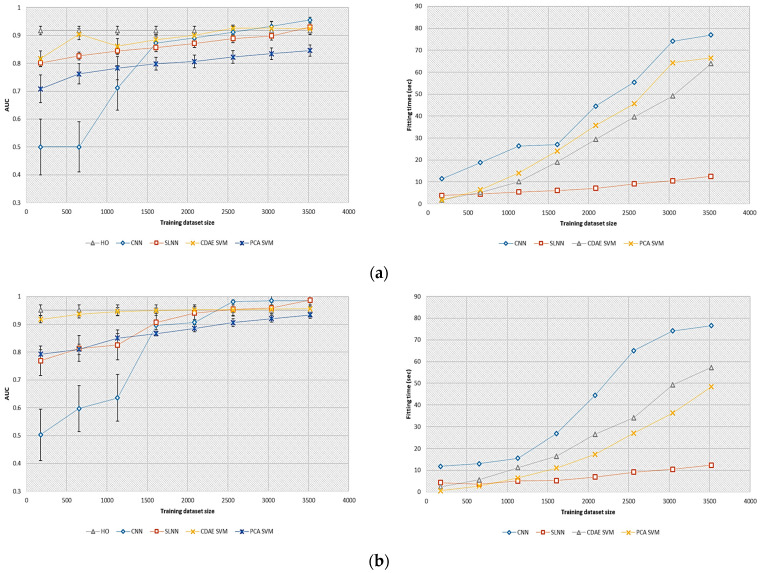
Effect of the training dataset size on detection performance and fitting times for the supervised learning-based model observers and SSL-based model observers investigated. (**a**) AUC (**left**) and fitting times (**right**) of the selected models for the SKE/BKS detection task; (**b**) AUC (**left**) and fitting times (**right**) of the selected models for the SKS/BKS detection task.

In these experiments, the detection performance of the selected models was evaluated on a reduced number of training images in order to be consistent with the realizations achievable in clinical practice. Figure 3 shows the AUC and fitting times obtained by the different observers investigated as a function of the training dataset size. We can note that a straight line was plotted to represent the HO performance, as it was estimated from a fixed training dataset.

In both tasks, the CDAE-based model observer provided nearly similar performance to the HO when using at least 1000 images. For the SKE/BKS task, which was more complex, this observer provided higher AUC values than the PCA-based model. This result demonstrated that the CDAE was able to extract more discriminative features for classification than the linear PCA. The CNN and SLNN provided higher detection performance than the HO when a sufficient amount of data was considered (i.e., at least 2500 images). It is worth mentioning that the CDAE-based model outperformed the supervised learning-based model observers on a reduced number of training images (i.e., less than 1000).

The storage requirement and computational complexity of the CNN were larger than those of the other models investigated. We can attribute this result to the number of variables estimated by the CNN, which was larger than that of the SLNN or SVM.

## 4. Discussion

The current state of SSL research has already achieved promising results in different domains of medicine such as digital pathology or computer-aided diagnosis [15,16,17,26]. In particular, the SSL paradigm can take advantage of the large volumes of unlabeled datasets available in medical imaging [6,15]. Thus, this approach may address the challenges impeding deep-learning models to become widespread in medical image analysis. Taking inspiration from the literature, this work adopted a two-stage model, which combined the CDAE network for feature extraction and SVM for the classification task, to produce accurate estimates of HO performance in low-dose chest CT images.

The proposed SSL-based model provided similar detection performance to the HO under various noise structures. This point is of interest because the ability of an observer to generalize well over different reconstruction algorithms is crucial to optimizing and standardizing a fleet of scanners [29,44]. We conjecture that the application of a regularization constraint during the CDAE training process gave this model a better generalization capability than that of the linear PCA. Although not presented in this paper, no direct correlation was observed between the AE reconstruction loss and the classification performance. This emphasizes the fact that the features learned by AE do not necessarily guarantee strong discriminative ability [22]. To address this concern, an alternative was proposed by several authors, which tends to simultaneously minimize the reconstruction loss of AE and the structural risk term of the classifier [22,25]. Such a framework imposes the encoder to project input data into discriminative subspaces for classification and remains a topic of future investigation [22].

The effectiveness of the model strongly depends on the SVM hyper-parameter selection. The results obtained demonstrated that the hyper-parameters have to be tuned with regard to the features learned in the pre-training step. The radius of the RBF kernel σ acted as a good regularizer regardless of the feature extraction technique experimented. Intermediate values of σ seemed suitable in order to prevent overfitting issue on one hand and avoid too “smooth” models on the other hand [43]. For these values of σ, the CDAE-based model was found to be unsensitive to changes in the values of the *C* parameter. Therefore, using small values of *C* allowed a decrease in the training and testing time, which is an important factor to consider for straightforward implementation.

Moreover, the proposed SSL-based model observer provided a higher detection performance than the state-of-the-art supervised learning-based methods when a reduced number of training images was considered. This result is consistent with previous published works, where the authors showed that a large amount of labeled data is required for implementing the CNN [3,6]. In this work, the addition of convolutional layers in the CNN architecture resulted in a decrease in the detection performance. An early-stopping strategy could be employed during the network training to reduce this overfitting problem. Alternatively, a multiple classifier that makes interim decisions instead of one at the end of the network would be used [45].

This study has several limitations. First, our experiments have focused on classification tasks in chest CT images. This work should be extended to other anatomical localizations and diagnostic tasks in order to demonstrate the generalizability of this method as a surrogate for the HO. Second, we intend to extend this work to handle the 3D regularization along both axial and longitudinal directions used by current reconstruction algorithms in CT images. Finally, we will evaluate the hierarchical feature extractor proposed by Ahn et al. [12,13], which introduced a CAE placed atop a pre-trained CNN, to further improve the feature representation of image data.

## 5. Conclusions

In summary, this study proposed an approach that combined deep and shallow learning techniques to match the performance of the HO for low-dose chest CT images. This approach leveraged a large amount of unlabeled data, which can be accessed through archives or shared databases, to learn meaningful representations for enhancing classification and generalization performance. This approach thus reduced the reliance on labeled data. Therefore, the proposed CDAE-based model observer may be easier to deploy than the HO for use as a task-based measure of image quality. We anticipate that the SSL-based approach can contribute to more accurate image quality analysis and can be a useful tool for objective comparison between different CT scanners or reconstruction algorithms.

## Figures and Tables

**Table 1:** Optimal values of *C* and *σ*, and the corresponding AUC for each detection task considered.

	*CDAE*				*PCA*
	λ = 0.00001	λ = 0.0001	λ = 0.001	λ = 0.01	
*C* *(SKE/BKS, SKS/BKS)*	10^4^, 10^3^	10^3^, 10^2^	10^1^, 10^2^	10^7^, 10^6^	10^3^, 10^2^
*σ* *(SKE/BKS, SKS/BKS)*	100, 100	10, 10	1, 1	0.01, 0.1	0.1, 0.1
*AUC (SKE/BKS, SKS/BKS)*	0.76, 0.74	0.77, 0.70	0.88, 0.91	0.63, 0.65	0.78, 0.86

## Data Availability

Dataset available on request from the authors.
